# Three new species of *Fomitiporella* (Hymenochaetales, Basidiomycota) based on the evidence from morphology and DNA sequence data

**DOI:** 10.3897/mycokeys.30.23109

**Published:** 2018-03-08

**Authors:** Xiao-Hong Ji, Josef Vlasák, Xue-Mei Tian, Yu-Cheng Dai

**Affiliations:** 1 Beijing advanced innovation center for tree breeding by molecular design, Beijing Forestry University, Beijing 100083, PR China; 2 Institute of Microbiology, PO Box 61, Beijing Forestry University, Beijing 100083, China; 3 Biology Centre of the Academy of Sciences of the Czech Republic, Branišovská 31, CZ-370 05 České Budějovice, Czech Republic; 4 Shandong Provincial Key Laboratory of Applied Mycology, Qingdao Agricultural University, Qingdao 266109, China

**Keywords:** Hymenochaetaceae, Polypore, Taxonomy, Phylogenetic analysis

## Abstract

*Fomitiporella austroasiana*, *F. mangrovei* and *F. vietnamensis* are described and illustrated as new species based on morphological characters and molecular evidence. They have annual to perennial, mostly resupinate basidiomata with grayish fresh pores, an indistinct subiculum, lack any kind of setae, have brownish, thick-walled basidiospores, and cause a white rot. The distinctive morphological characters of the new species and their related species are discussed. Phylogenies based on the nuclear ribosomal large subunit (28S) and the nuclear ribosomal ITS region show that these three new species form three distinct lineages in the *Fomitiporella* clade. A key to known species of *Fomitiporella* is given.

## Introduction

*Fomitiporella* Murrill was described by Murrill (1907) with *F. umbrinella* as type. The genus is characterized by perennial, resupinate and adnate basidiomata, a thin subiculum, stratified tubes, and brown, subglobose basidiospores (Murrill 1907). *Fomitiporella* has been considered a synonym of *Phellinus* (Ryvarden and Johansen 1980, Larsen and Cobb-Poulle 1990, Ryvarden 1991, Ryvarden and Gilbertson 1994, Dai 1999, Núñez and Ryvarden 2000). A previous phylogenetic study based on 28S DNA sequence data confirmed *Fomitiporella* as an independent genus within Hymenochaetaceae, with *Phellinus caryophyllii* (Racib.) G. Cunn. and *P. cavicola* Kotl. & Pouzar transferred into *Fomitiporella* (Wagner and Fischer 2002). During the past five years, many new species were revealed based on morphological characters and molecular data (Zhou 2014, Ji et al. 2017). Recently, Ji et al. (2017) broadened the concept of *Fomitiporella* to accommodate species with resupinate to effused reflexed and annual basidiomata.

As a continuation of the revision of *Fomitiporella* Murrill, phylogenetic inferences based on 28S and ITS DNA sequences revealed three new species. The taxonomic affinity and the evolutionary relationships among the new species and relates species are outlined.

## Materials and methods

### Morphological studies

Specimens studied are deposited in the herbarium of Beijing Forestry University (BJFC) and will be forwarded to the National Museum Prague of Czech Republic (PRM). The sections were prepared in 5% potassium hydroxide (KOH), Melzer’s reagent (IKI) and Cotton Blue (CB). The following abbreviations were used: KOH = 5% potassium hydroxide, IKI = Melzer’s reagent, IKI– = neither amyloid nor dextrinoid, CB = Cotton Blue, CB+ = cyanophilous, CB(+) = cyanophilic after 12 hours stained with Cotton Blue, CB– = acyanophilous, L = mean spore length (arithmetic average of the spores), W = mean spore width (arithmetic average of the spores), Q = variation in the ratios of L/W between specimens studied and n = number of spores measured from new specimens. The microscopic procedure follows He and Li (2013) and the special color terms follow Petersen (1996). Sections were studied at magnifications up to 1000× using a Nikon Eclipse 80i microscope with phase contrast illumination. Drawings were made with the aid of a drawing tube. Microscopic features, measurements, and illustrations were made from slide preparations stained with Cotton Blue. Spores were measured from sections cut from the tubes.

### Molecular study and phylogenetic analysis

A CTAB-based rapid plant genome extraction kit (Aidlab Biotechnologies Co., Ltd, Beijing) was used to obtain genomic DNA from dried specimens. The primer pair ITS4 and ITS5 was used for amplification of the ITS region (White et al. 1990), while the primer pair LR0R and LR7 (http://www.biology.duke.edu/fungi/mycolab/primers.htm) was used for providing the D1–D4 regions of the 28S (https://unite.ut.ee/primers.php). The PCR procedure for ITS amplification was as follows: initial denaturation at 95°C for 3 min, followed by 35 cycles at 94 °C for 40 s, 54 °C for 45 s and 72 °C for 1 min, and a final extension of 72 °C for 10 min. The PCR procedure for 28S was as follows: initial denaturation at 94 °C for 1 min, followed by 35 cycles at 94 °C for 30 s, 50 °C for 1 min and 72 °C for 1.5 min, and a final extension of 72 °C for 10 min. The PCR products were purified and sequenced at the Beijing Genomics Institute, China, with the same primers.

Reference ITS and 28S sequences from various species of *Fomitiporella*, available from GenBank (Benson et al. 2017), were compiled and complemented with sequences generated for this study. Additionally, we also used sequences from Ji et al. (2017) (Table 1). *Phellinus laevigatus* (P. Karst.) Bourdot & Galzin and *P. populicola* Niemelä were selected as the outgroup representatives both in the ITS dataset and 28S dataset (Wagner and Fischer 2002). The sequences were aligned using ClustalX 1.83 (Chenna et al. 2003) and alignments were curated manually in BioEdit 7.0.5.3 (Hall 1999). Prior to phylogenetic analyses, ambiguous regions at the start and the end were deleted. The sequence alignment was deposited at TreeBase (submission ID 22036; www.treebase.org). Phylogenetic analyses were carried out as described previously (Ji et al. 2017).

**Table 1.** Information on the sequences used in this study. Type specimens are shown in bold.

Species	Location	Sample no.	GenBank accession no.	
			ITS	28S
*Fomitiporella americana*	USA	JV 0312/26.6J	KX181291	–
*F. americana*	USA	JV 0212/8J	KX181292	–
*F. americana*	USA	JV 0904/149J	KX181293	KX181329
***F. austroasiana***	**China**	**Dai 16244**	**MG657328**	**MG657320**
*F. austroasiana*	China	Dai 16168	MG657329	MG657321
*F. austroasiana*	Singapore	Dai 17868	–	MG657322
*F. austroasiana*	Singapore	Dai 17871	–	MG657323
*F. austroasiana*	Singapore	Dai 17879	MG657330	MG657324
*F. caryophyllii*	India	CBS 448.76	AY558611	AY059021
*F. cavicola*	UK	N 153	–	AY059052
*F. caviphila*	China	LWZ 20130812-1	–	KF729937
*F. chinensis*	China	Cui 11097	KX181310	KX181342
*F. chinensis*	China	Cui 11091	–	KX181340
*F. chinensis*	China	LWZ 20130713-7	KJ787817	KJ787808
*F. chinensis*	China	LWZ 20130916-3	KJ787818	KJ787809
*F. chinensis*	China	Cui 11095	–	KX181341
*F. chinensis*	China	Cui 8725	–	KX181343
*F. inermis*	USA	JV 0509/57K	KX181305	KX181346
*F. inermis*	USA	JV 1109/19A	KX181304	–
*F. inermis*	USA	JV 1009/56	KX181306	KX181347
***F. mangrovei***	**USA**	**JV 1008/60**	**KX181313**	**KX181334**
*F. mangrovei*	France	JV 1612/25-J	MG657331	MG657325
*F. micropora*	USA	JV 1312/E2J	KX181294	KX181333
*F. micropora*	USA	JV 1407/46	KX181295	KX181332
*F. micropora*	USA	JV 0409/6J	KX181296	KX181331
*F. micropora*	USA	JV 1207/6.1J	KX181297	KX181330
*F. resupinata*	Cameroon	Douanla-Meli 476	KJ787822	JF712935
*F. sinica*	China	Cui 10139	KX181298	–
*F. sinica*	China	Dai 10461	KX181300	–
*F. sinica*	China	LWZ 20130809-8	KJ787820	KJ787811
*F. sinica*	China	LWZ 20140625-2	KX181301	KX181320
*F. sinica*	China	LWZ 20140624-5	KX181302	KX181321
*F. sinica*	China	Dai 12450	–	KX181326
*F. sinica*	China	Dai 13944	–	KX181324
*F.* sp. 1	China	Cui 6557	KX181303	–
*F.* sp. 2	China	Cui 11352	KX181315	KX181338
*F.* sp. 3	China	LWZ 20140721-2	KX181316	KX181337
*F.* sp. 4	Thailand	LWZ 20140729-22	KX181317	KX181339
*F.* sp. 5	Chile	Fv.Ch-7	–	DQ459301
*F.* sp. 6	Ethiopia	AM 12	JF895466	JQ910908
*F.* sp. 7	Ethiopia	AM 15	JF895467	JQ910909
*F.* sp. 8	Ethiopia	AM 18	JF895468	JQ910910
*F.* sp. 9	Ethiopia	AM 04	KX181318	KX181335
*F. subinermis*	China	Dai 15114	KX181308	KX181344
*F. subinermis*	China	Dai 15131	KX181307	KX181345
*F. tenuissima*	China	Dai 12365	KC456244	KC999901
*F. tenuissima*	China	Dai 12245	KC456242	KC999902
*F. tenuissima*	China	Dai 12255	KC456243	KC999903
*F. tenuissima*	China	Cui 10960	KX181319	–
*F. umbrinella*	USA	0509/114	KX181314	KX181336
*F. umbrinella*	USA	CBS 303.66	–	AY059036
***F. vietnamensis***	**Vietnam**	**Dai 18377**	**MG657332**	**MG657326**
*F. vietnamensis*	Vietnam	Dai 18382	MG657333	MG657327
*Fulvifomes fastuosus*	Thailand	LWZ 20140801-1	KR905675	KR905669
*F. robiniae*	USA	CBS 211.36	AY558646	AF411825
*Inonotus hispidus*	Germany	MF 92-829	–	AF311014
*I. hispidus*	–	CBS 386.61	AY558602	AY558664
*I. obliquus*	Germany	TW 705	–	AF311017
*I. quercustris*	Argentina	0193	AY072026	AY059050
*I. andersonii*	USA	CBS 312.35	–	AY059041
*Phylloporia bibulosa*	Pakistan	Ahmad 27088	–	AF411824
*P. chrysites*	Puerto Rico	N.W. Legon	–	AF411821
*P. ephedrae*	Turkmenistan	TAA 72-2	–	AF411826
*P. pectinata*	UK	R. Coveny 113	–	AF411823
*P. ribis*	Germany	MF 82-828	–	AF311040
*P. spathulata*	Mexico	Chay 456	–	AF411822
*Phellinus laevigatus*	Finland	TN 3260	–	AF311034
*P. laevigatus*	–	83-912	AY340051	–
*P. populicola*	Germany	MF 84-61	–	AF311038
*P. populicola*	Sweden	BRNM 714885	GQ383706	–

Maximum likelihood (ML), maximum parsimony (MP) and Bayesian inference (BI) analyses were performed for the two datasets. The three phylogenetic analysis algorithms generated nearly identical topologies for each dataset, thus only the topology from the MP analysis is presented along with statistical values from the ML, MP and BI algorithms (Bootstrap support < 50 % and Bayesian posterior probabilities < 0.9 are not shown) at the nodes. MP analyses were performed using PAUP* 4.0b10 (Swofford 2002) with gaps in the alignments treated as missing data. Trees were generated using 100 replicates of random stepwise addition of sequence and tree-bisection reconnection (TBR) branch-swapping algorithm with all characters given equal weight. Branch supports (BS) for all parsimony analyses were estimated by performing 1,000 bootstrap replicates (Felsenstein 1985) with a heuristic search of 10 random-addition replicates for each bootstrap replicate. Sequences were also analyzed using MLwith RAxML-HPC2 on Abe through the Cipres Science Gateway (www.phylo.org). BI was calculated with MrBayes3.1.2 with a general time reversible (GTR) model of DNA substitution and a gamma distribution rate variation across sites (Ronquist and Huelsenbeck 2003). The ITS region was divided into three partitions, ITS1, 5.8S and ITS2, for the Bayesian analysis. MrModeltest2.3 (Posada and Crandall 1998, Nylander 2004) was used to determine the best-fit evolution model for each dataset. Trees were visualized in TreeView 1.6.6 (Page 1996).

## Results

Fifty-six 28S rDNA sequences, including eight sequences generated in this study (GenBank accession numbers MG657320–MG657327) and forty-six ITS rDNA sequences, including six sequences generated in this study (GenBank accession numbers MG657328–MG657333) were used to infer the phylogenetic trees. Sequence information is provided in Table 1. The 28S dataset had an aligned length of 898 characters, of which 628 characters are constant, 84 are variable and parsimony-uninformative, and 186 (21%) are parsimony-informative. The best-fit model for the 28S dataset estimated and applied in the Bayesian analysis: GTR+I+G, lset nst = 6, rates = invgamma; prset statefreqpr = dirichlet (1,1,1,1). The ITS dataset had an aligned length of 854 characters, of which 350 are constant, 114 variable and parsimony-uninformative, and 390 (46 %) parsimony-informative. The best-fit models for the three partitions estimated and applied in the Bayesian analysis are as follows: HKY+I+G for ITS1, K80+I+G for 5.8S and HKY+G for ITS2. The Bayesian and ML analyses produced similar topologies compared to the MP analysis, with an average standard deviation of split frequencies = 0.006943 (BI) (28S). Bayesian analysis and ML analysis resulted in a similar topology as the MP analysis, with an average standard deviation of split frequencies = 0.009677 (BI) (ITS).

**Figure 1.**
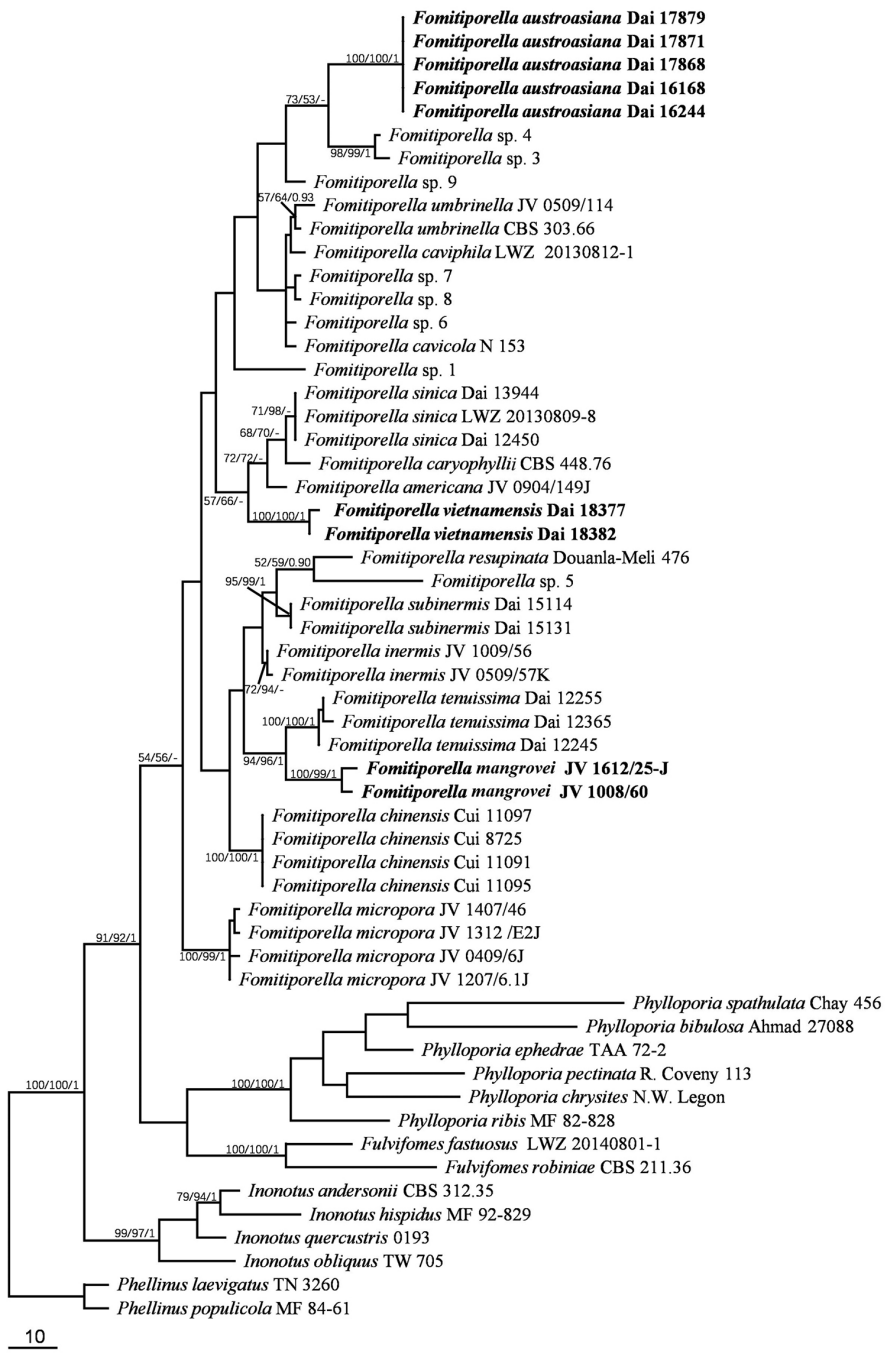
Phylogeny of *Fomitiporella* inferred from the 28S dataset. The topology is that of the MP analysis, and statistical values (ML/MP/BI) are indicated for each node that simultaneously received BS from ML and MP not below 50 %, and BPP from BI not below 0.9. *Phellinus laevigatus* and *P. populicola* are used to root the tree. Branch lengths reflect the number of steps as indicated by the scale.

**Figure 2.**
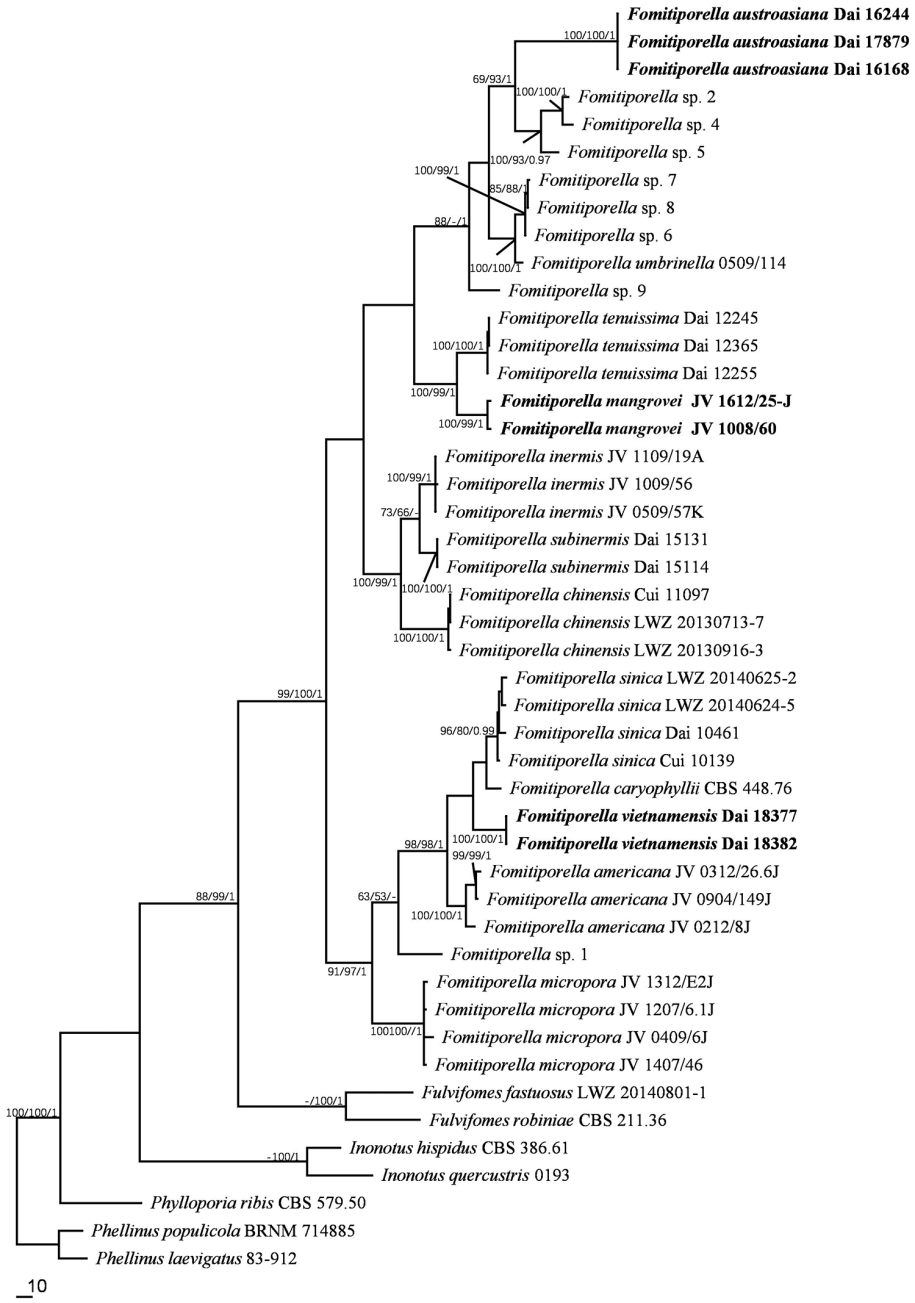
Phylogeny of *Fomitiporella* inferred from the ITS dataset. The topology is that of the MP analysis, and statistical values (ML/MP/BI) are indicated for each node that simultaneously received BS from ML and MP not below 50 %, and BPP from BI not below 0.9. *Phellinus laevigatus* and *P. populicola* are used to root the tree. Branch lengths reflect the number of steps as indicated by the scale.

The current phylogenies (Figs 1, 2) confirmed that *Fomitiporella austroasiana*, *F. mangrovei* and *F. vietnamensis* formed three strongly supported clades (all received strong branch support in the ML, BI and MP analyses). These taxa have typical morphology of the current concept of *Fomitiporella* (Ji et al. 2017). However, each clade has its unique characters distinct from other *Fomitiporella* species. We therefore describe them as new species.

## Taxonomy

### Fomitiporella austroasiana

Taxon classificationFungiORDOFAMILIA

Y.C. Dai, X.H. Ji & J. Vlasák sp. nov.

MB823738

Figs 3 , 4

#### Holotype.

CHINA. Hainan Province: Qiongzhong County, Limushan Forest Park, 15 Nov 2015, on fallen angiosperm trunk, *Dai 16244* (BJFC).

**Figure 3.**
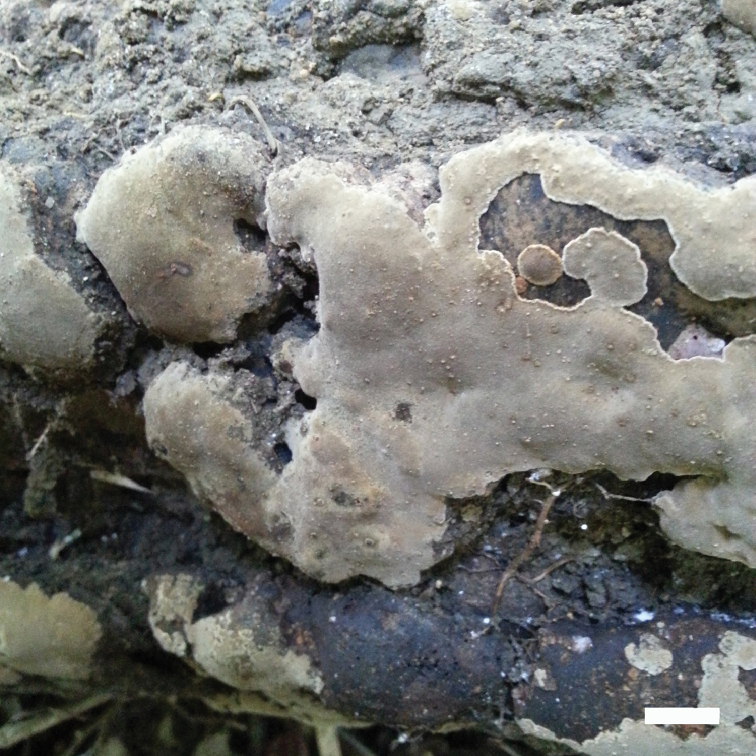
A basidiocarp of *Fomitiporella austroasiana*. Scale bar: 1 cm.

**Figure 4.**
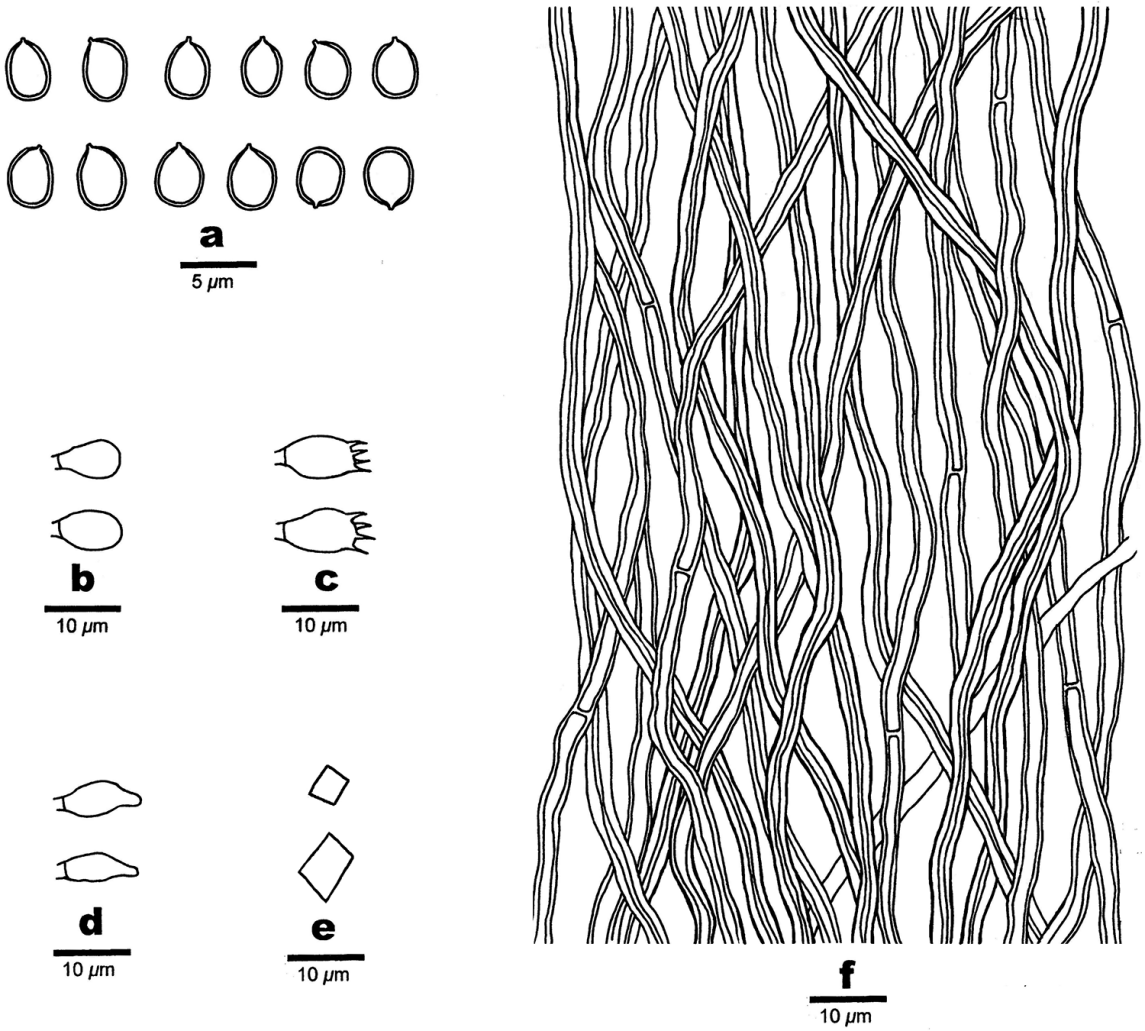
Microscopic structures of *Fomitiporella austroasiana*. **a** Basidiospores **b** Basidioles **c** Basidia **d** Cystidioles **e** Rhomboid crystals **f** Hyphae from trama.

**Etymology**. *Austroasiana* (Lat.): referring to the distribution of the species in South Asia.

Basidiomata perennial, resupinate, hard corky and without odor or taste when fresh, woody hard when dry, up to 12 cm long, 5 cm wide and 12 mm thick at center. Pore surface ash-gray to grayish brown when fresh, grayish brown to olivaceous, more or less shiny and uncracked on drying; margin yellowish-brown, less than 1 mm wide, thinning out; pores circular, 8–10 per mm; dissepiments thick, entire; tubes woody hard, concolorous with pores, each layer up to 2 mm deep, white mycelial strands present in old tubes. Subiculum very thin to almost lacking.

#### Hyphal structure.

Hyphal system dimitic; generative hyphae simple septate; skeletal hyphae dominant; tissue darkening but otherwise unchanged in KOH.

#### Tubes.

Generative hyphae frequent, hyaline to pale yellow, thin- to slightly thick-walled, occasionally branched, frequently simple septate 1.5–2.5 μm in diam; skeletal hyphae pale brown to brown, thick-walled to almost solid, aseptate, 2–3 μm in diam; setae absent; cystidioles ventricose with elongated apical portion, 7–12 × 3–4 µm; basidia barrel-shaped, with four sterigmata and a simple basal septum, 8–11 × 5–6 μm; basidioles similar to basidia in shape, but slightly smaller; small or big rhomboid crystals abundant.

#### Spores.

Basidiospores subglobose, yellowish-brown, thick-walled, IKI–, CB(+), (3.5–)3.8–4(–4.3) × 3–3.5 μm, L = 4 μm, W = 3.29 μm, Q = 1.2–1.21 (n = 60/2).

#### Additional specimens examined (paratypes).

CHINA. Hainan Province: Wuzhishan, Wuzhishan Nature Reserve, 14 Nov 2015, on fallen angiosperm trunk, *Dai 16168* (BJFC). SINGAPORE. Bukit Timah Nature Reserve, 20 June 2017, *Dai 17868*; *Dai 17871*; *Dai 17879* (BJFC).

### Fomitiporella mangrovei

Taxon classificationFungiORDOFAMILIA

Y.C. Dai, X.H. Ji & J. Vlasák sp. nov.

MB823743

Figs 5 , 6

#### Holotype.

USA. Florida: Collier-Seminole State Park, 28 Aug 2010, on *Conocarpus erectus*, *JV 1008/60* (BJFC).

**Figure 5.**
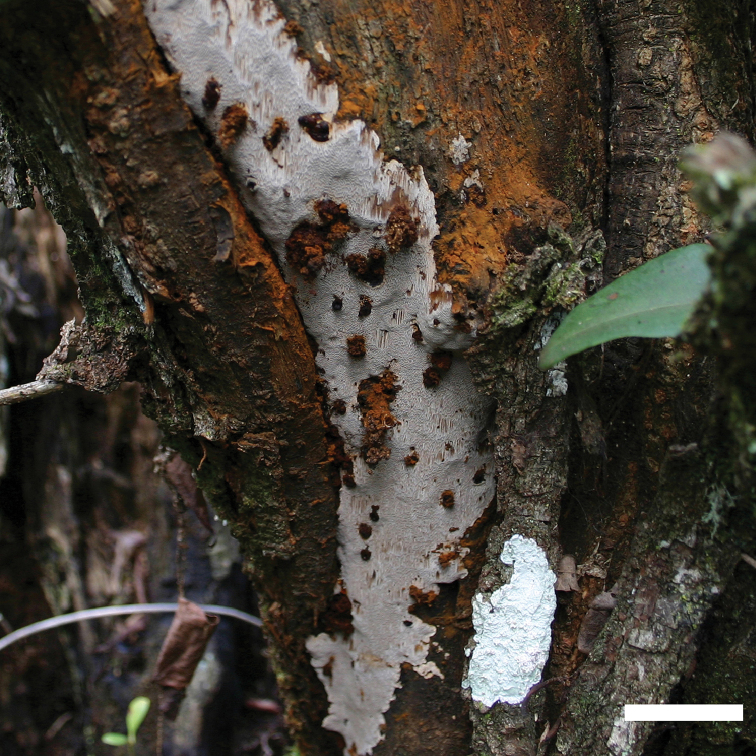
Basidiomata of *Fomitiporella mangrovei*. Scale bar: 5 cm.

**Figure 6.**
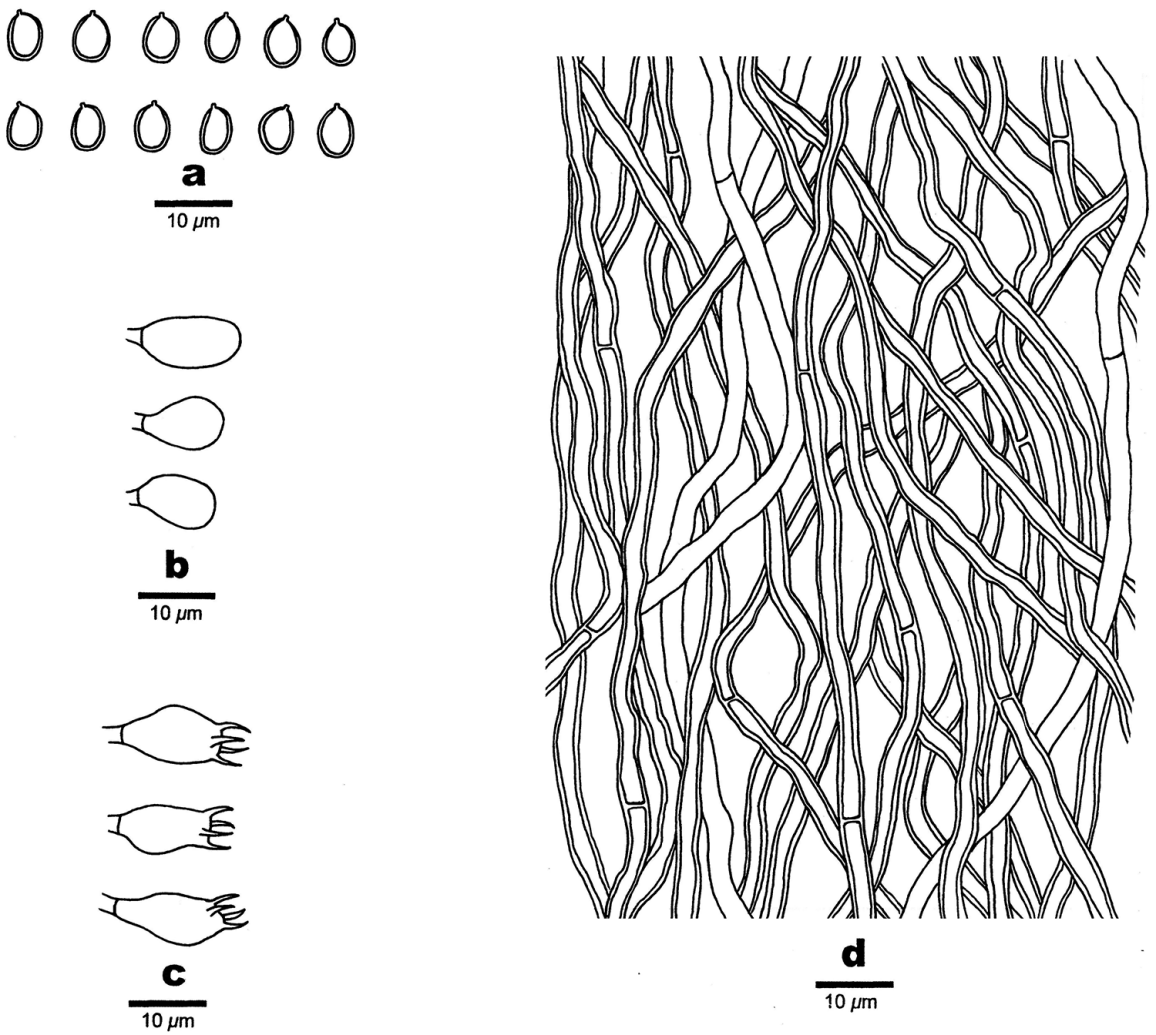
Microscopic structures of *Fomitiporella mangrovei*. **a** Basidiospores **b** Basidioles **c** Basidia **d** Hyphae from trama.

#### Etymology.

*Mangrovei* (Lat.): referring to the species growing in mangrove. Basidiomata annual, resupinate, inseparable, without odor or taste when fresh, woody hard on drying, up to 30 cm long, 7 cm wide and 5 mm thick at center. Pore surface ash-gray to bluish gray when fresh, becomes pale clay-buff to pale brown and uncracked when dry; pores angular, 3–5 per mm; dissepiments thin, more or less entire to slightly lacerate; tubes woody hard, dark brown, up to 5 mm long. Subiculum very thin to almost lacking.

#### Hyphal structure.

Hyphal system monomitic; generative hyphae simple septate; tissue darkening but otherwise unchanged in KOH.

#### Tubes.

Generative hyphae hyaline to pale yellowish, thin- to thick-walled with a wide lumen, occasionally branched, frequently simple septate, interwoven, 1.5–3 mm in diam; setae absent; cystidioles absent; basidia barrel-shaped, with four sterigmata and a simple basal septum, 12–15 × 4–6 μm; basidioles barrel-shaped to pyriform, slightly smaller than basidia in size.

#### Spores.

Basidiospores broadly ellipsoid, yellowish-brown, thick-walled, smooth, IKI–, CB+, (5–)5.5–6(–6.3) × (4–)4.2–4.8(–5) μm, L = 5.82 μm, W = 4.47 μm, Q = 1.26–1.31 (n = 60/2).

#### Additional specimen examined (paratype).

FRANCE. Guadeloupe: Grande-Terre, 25 Dec 2012, on *Conocarpus erectus*, *JV 1612/25-J* (BJFC).

### Fomitiporella vietnamensis

Taxon classificationFungiORDOFAMILIA

Y.C. Dai, X.H. Ji & J. Vlasák sp. nov.

MB823744

Figs 7 , 8

#### Holotype.

VIETNAM. Lam Dong Province, Lac Duong District, Bidoup Nui Ba National Park, 15 Oct 2017, on angiosperm tree, *Dai 18377* (BJFC).

**Figure 7.**
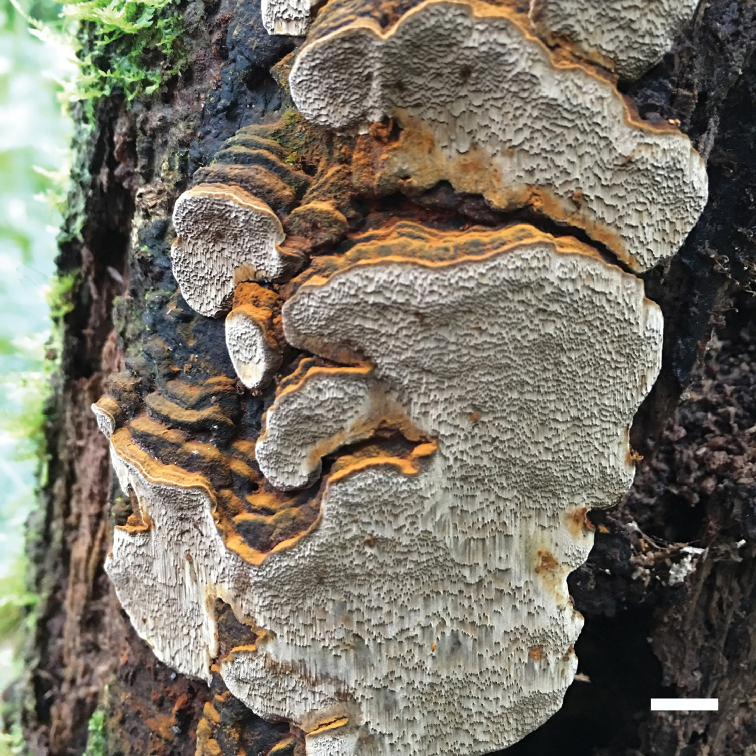
Basidiomata of *Fomitiporella vietnamensis*. Scale bar: 1 cm.

**Figure 8.**
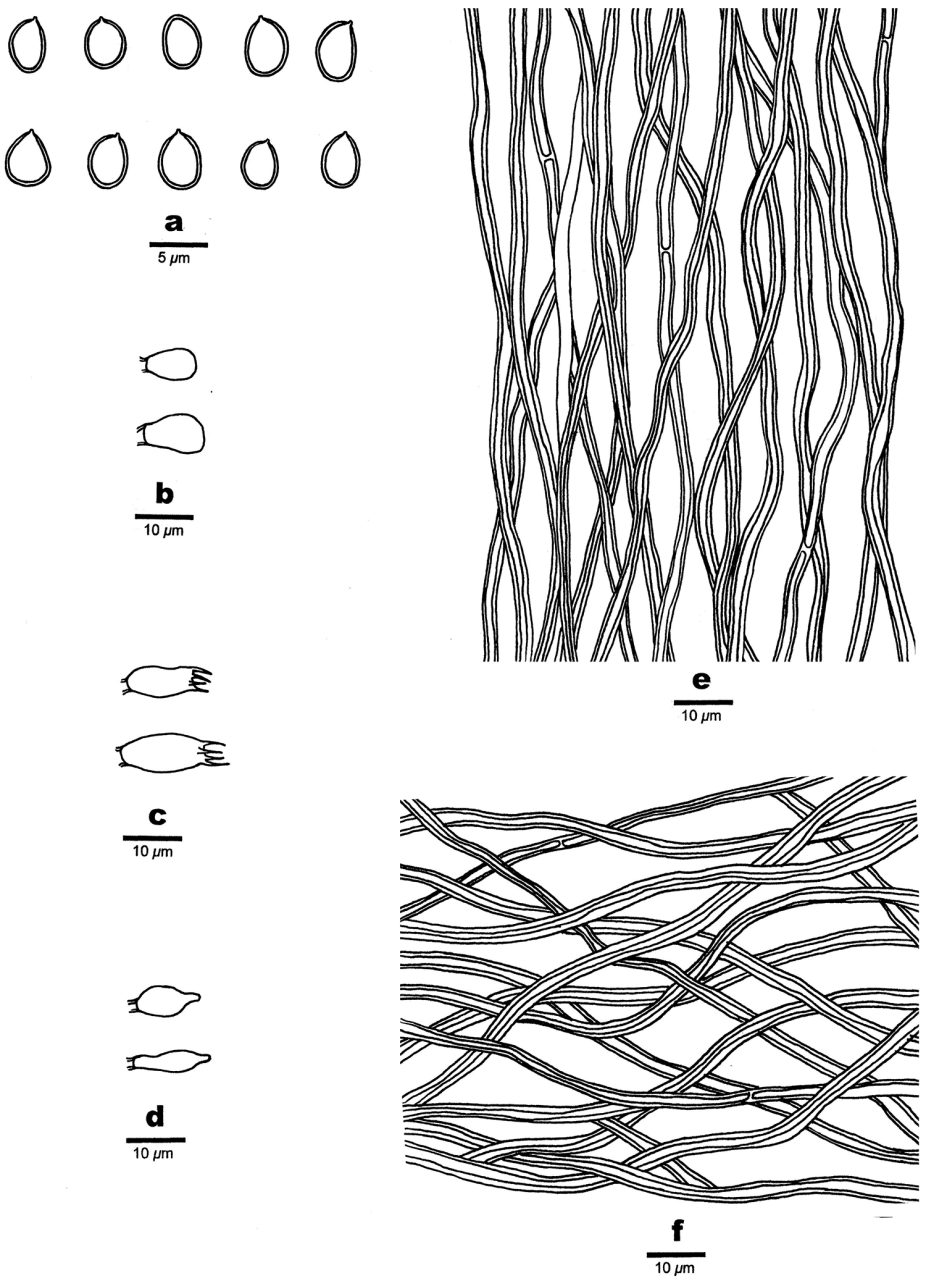
Microscopic structures of *Fomitiporella vietnamensis*. **a** Basidiospores **b** Basidioles **c** Basidia **d** Cystidioles **e** Hyphae from trama **f** Hyphae from subiculum.

#### Etymology.

*Vietnamensis* (Lat.): referring to the distribution of the species in Vietnam.

Basidiomata perennial, effused-reflexed, imbricate, hard corky and without odor or taste when fresh, projecting up to 1 cm long, 4 cm wide and 5.5 mm thick. Pileal surface bearing curry-yellow and black zones when fresh, becoming deep olive when dry; pore surface bluish gray to ash-gray when fresh, becomes dark brick, shiny and uncracked on drying; margin yellowish-brown, less than 1 mm wide, thinning out; pores angular to circular, 4–7 per mm; dissepiments thin, slightly lacerate. Tubes rust-brown, paler contrasting with pores, up to 5 mm long. Subiculum dull brown, hard corky, up to 0.5 mm.

#### Hyphal structure.

Hyphal system dimitic; generative hyphae simple septate; skeletal hyphae dominant; tissue darkening but otherwise unchanged in KOH.

#### Subiculum.

Generative hyphae rare, hyaline to pale yellowish, thick-walled, rarely branched and septate, 2–2.5 µm in diam; skeletal hyphae dominant, golden yellow, thick-walled with a wide lumen, unbranched, aseptate, more or less flexuous, interwoven, 2–3.5 µm in diam.

#### Tubes.

Generative hyphae frequent, hyaline to pale yellowish, thin- to fairly thick-walled, occasionally branched, frequently septate, 2–2.7 µm in diam; skeletal hyphae dominant, golden yellow, thick-walled, unbranched, aseptate, straight, more or less parallel along the tubes, 2–3 µm in diam; setae absent; cystidioles ventricose with elongated apical portion, 7–14 × 3–5 µm; basidia barrel-shaped, with four sterigmata and a simple basal septum, 10–16 × 5–6 μm; basidioles similar to basidia in shape, but slightly smaller.

#### Spores.

Basidiospores broadly ellipsoid, yellowish-brown, thick-walled, IKI–, CB+, 4–4.8(–5) × (3–)3.2–3.7(–4) μm, L = 4.41 μm, W = 3.52 μm, Q = 1.23–1.28 (n = 60/2).

#### Additional specimen examined (paratype).

VIETNAM. Lam Dong Province, Lac Duong District, Bidoup Nui Ba National Park, 15 Oct 2017, on angiosperm tree, *Dai 18382* (BJFC).

## Discussion

*Fomitiporella austroasiana* fits well in *Fomitiporella* (redefined in Ji et al. 2017). In the current phylogenies (Figs 1, 2), *F. austroasiana* forms a new, strongly supported clade. Macroscopically, *F. austroasiana* is similar to *F. micropora* Y.C. Dai, X.H. Ji & Vlasák in sharing perennial, resupinate basidiomata and small pores (8–10 per mm), a dimitic hyphal structure, and slightly cyanophilous basidiospores (3–4.5 × 2–3.5 μm), whereas *F. micropora* has ellipsoid basidiospores (Q=1.27–1.3, Ji et al. 2017). Moreover, the presence of the cystidioles in *F. austroasiana* makes it different from *F. micropora*.

*Fomitiporella mangrovei* was previously treated as an undescribed taxon (*Fomitiporella* sp.1) because only a single collection from Florida (USA) was available (Ji et al. 2017). Another specimen, collected from Guadeloupe, Lesser Antilles, was found to represent the same taxon, allowing a better description. *Fomitiporella mangrovei* is characterized by annual, resupinate basidiomata with ash-gray to bluish gray pores when fresh, large pores (3–5 per mm), a monomitic hyphal structure, ellipsoid, yellowish and thick-walled basidiospores (5–6.3 × 4–5 μm), and growing on *Conocarpus erectus* (Combretaceae), in mangrove ecosystem. Macroscopically it resembles *F. tenuissima* (H.Y. Yu, C.L. Zhao & Y.C. Dai) Y.C. Dai, X.H. Ji & J. Vlasák and the species are closely related (Figs 1, 2), but *F. tenuissima* differs in having smaller basidiospores (4–5 × 3–4 μm; Yu et al. 2013).

*Fomitiporella vietnamensis* is distinct by a combination of perennial, effused-reflexed and imbricate basidiomata, shiny and uncracked pore surface, a dimitic hyphal system, and broadly ellipsoid basidiospores, 4–5 × 3–4 μm. *Fomitiporella vietnamensis* is closely related to *F. caryophyllii* (Racib.) T. Wagner & M. Fisch in the current phylogenies (Figs 1, 2). Morphologically, both species share the perennial, effused-reflexed basidiomata and a dimitic hyphal system (Ryvarden and Johansen 1980). However, *F. caryophyllii* has smaller pores (7–9 per mm) and smaller basidiospores of 3–4 × 2.5–3 μm (Ryvarden and Johansen 1980). Another species close to *F. vietnamensis* is *F. americana* Y.C. Dai, X.H. Ji & J. Vlasák (Figs 1, 2), but *F. americana* has strictly resupinate basidiomata and lacks cystidioles (Ji et al. 2017).

The phylogenetic analyses based on 28S or the ITS dataset produced trees with near-identical topologies, and each of the three new species formed a distinct, well-supported clade.

An identification key to the accepted species of *Fomitiporella* is provided as follows:

### Key to species of *Fomitiporella*

**Table:** 

1	Basidiocarp pileate to effused-reflexed	**2**
–	Basidiocarp resupinate	**4**
2	Pores 3–7 per mm; basidiospores > 4 µm long	**3**
–	Pores 7–9 per mm; basidiospores < 4 µm long	***F. caryophyllii***
3	Basidiomata biennial; pores 3–4 per mm; basidiospores mostly > 4.5 µm long	***F. chinensis***
–	Basidiomata perennial; pores 4–7 per mm; basidiospores mostly < 4.5 µm long	***F. vietnamensis***
4	Basidiomata annual; pore surface more or less grayish when fresh	**5**
–	Basidiomata perennial; pore surface brown when fresh	**6**
5	Pore surface vinaceous gray when fresh; basidiospores < 5 µm long	***F. tenuissima***
–	Pore surface ash-gray to bluish gray when fresh; basidiospores > 5 µm long	***F. mangrovei***
6	Cystidioles present	**7**
–	Cystidioles absent	**9**
7	Pores 5–7 per mm; basidiospores mostly > 4.5 µm long	**8**
–	Pores 8–10 per mm; basidiospores < 4.5 µm long	***F. austroasiana***
8	Basidiomata up to 3 mm thick at center; basidiospores broadly ellipsoid	***F. inermis***
–	Basidiomata up to 10 mm thick at center; basidiospores subglobose	***F. subinermis***
9	Pores 5–6 per mm	**10**
–	Pores 6–10 per mm	**11**
10	Basidiospores 4.7–5.5 µm long; growth mostly on *Fagus*	***F. cavicola***
–	Basidiospores 3.6–4.6 µm long; growth mostly on *Quercus*	***F. americana***
11	Basidiospores ≤ 4 µm long	***F. resupinata***
–	Basidiospores ≥ 4 µm long	**12**
12	Pores 6–8 per mm	**13**
–	Pores 8–10 per mm	***F. micropora***
13	Basidiospores broadly ellipsoid to subglobose, CB(+)	**14**
–	Basidiospores ellipsoid to broadly ellipsoid, CB–	***F. umbrinella***
14	Basidiospores < 4.5 µm long in average	***F. sinica***
–	Basidiospores > 4.5 µm long in average	***F. caviphila***

## Supplementary Material

XML Treatment for Fomitiporella austroasiana

XML Treatment for Fomitiporella mangrovei

XML Treatment for Fomitiporella vietnamensis
